# S2DA-GO: enhancing protein function prediction via gradient-decoupled cross-attention and semantic priors

**DOI:** 10.3389/fgene.2026.1880976

**Published:** 2026-07-09

**Authors:** Hailong Wang, Fujun Xiang, Jin Zhang, Tingjie Wu, Dong Wang, Qiang Wang, Bin Lu, Feifei Fu

**Affiliations:** 1 Yanzhao Electric Power Laboratory, North China Electric Power University, Baoding, China; 2 Department of Computer, North China Electric Power University, Baoding, China; 3 Engineering Research Center of Intelligent Computing for Complex Energy Systems, Ministry of Education, Baoding, China; 4 Hebei Key Laboratory of Knowledge Computing for Energy & Power, Baoding, China

**Keywords:** convolutional neural network, cross-attention, deep learning, gene ontology, protein function, protein sequence

## Abstract

Accurate automated protein function prediction is essential for bridging the widening gap between the exponential accumulation of uncharacterized protein sequences and the limited repository of experimentally verified functional annotations. However, despite recent advances in incorporating Gene Ontology (GO) priors and sequence-label interactions, existing computational methods still face substantial challenges in long-tailed multi-label settings, particularly in handling optimization instability caused by rare-label noise and in learning reliable representations for sparsely annotated GO terms. To address these challenges, we propose S2DA-GO, a sequence-based model using pre-trained protein language model embeddings and GO textual semantic priors for protein function prediction. S2DA-GO integrates a global contextual stream with a local target-aware stream to capture multi-scale functional patterns. To alleviate optimization instability caused by long-tailed label noise, we introduce a Gradient-Decoupled Cross-Attention Module (GDCAM), which reduces the interference of label-specific gradients on the shared backbone. In addition, we incorporate learnable residual semantic priors derived from BioBERT-encoded GO definitions, enhancing the model’s adaptability to rare functional terms. On the benchmark dataset, S2DA-GO outperformed the strong baseline GDTGO across all three GO branches, achieving notable relative AUPR improvements of 4.0% Molecular Function (MF), 6.0% Biological Process (BP), and 8.6% Cellular Component (CC), with AUPR scores reaching 65.1%, 33.4%, and 41.7%, respectively. Notably, S2DA-GO remained robust in low-homology settings and provided interpretable residue-level signals that accurately aligned with experimentally verified binding sites. Overall, S2DA-GO alleviates feature interference and improves prediction for sparsely annotated GO terms, providing a promising framework for large-scale annotation of uncharacterized proteins.

## 1 Introduction

Proteins are central executors of biological processes, participating in enzymatic catalysis, molecular transport, signal transduction, and cellular regulation (Lee et al., 2007). Accurate protein function annotation is therefore essential for understanding cellular mechanisms, identifying disease-associated biomarkers, discovering therapeutic targets, and advancing precision medicine (Jiang et al., 2016). Despite the recent success of deep learning in modeling other complex biomarkers, such as RNAs and disease associations (Wang et al., 2025a; 2025b; 2026), the experimental function characterization through biochemical assays, such as gene knockout, site-directed mutagenesis, or other *in vitro* and *in vivo* validation approaches, remains labor-intensive, time-consuming, and difficult to scale. Meanwhile, the rapid expansion of high-throughput sequencing has produced a vast number of uncharacterized protein sequences, creating a persistent gap between sequence availability and reliable functional annotation (Bateman et al., 2021). This gap has made automated protein function prediction (AFP) a central task in bioinformatics and computational biology.

Gene Ontology (GO)-based protein function prediction (Ashburner et al., 2000) has become one of the prevailing paradigms for AFP. GO provides a standardized functional vocabulary across three major branches: Molecular Function (MF), Biological Process (BP), and Cellular Component (CC). Since a protein may be associated with multiple GO terms, GO-based function prediction is naturally formulated as a large-scale multi-label classification problem. This task is further complicated by hierarchical dependencies among GO terms, complex inter-label correlations, and highly imbalanced label distributions (Valentini, 2011), where many functional terms are associated with only a limited number of annotated proteins.

To address this persistent annotation gap, computational AFP methods have generally evolved through three primary paradigms: homology-based inference, traditional machine learning, and deep learning architectures (Bonetta and Valentino, 2020; Boadu et al., 2025). Initially, homology-based methods operated on the evolutionary premise that sequence similarity implies functional conservation. Foundational tools such as BLAST (Altschul et al., 1990) infer protein functions through heuristic local sequence alignment against annotated databases. To capture functional conservation at the domain level, methods like FunFams (Das et al., 2015) classify sequences into structurally and functionally coherent families within the CATH framework (Orengo et al., 1997). However, the efficacy of these alignment-driven approaches drops when the low sequence identity, rendering them inadequate for highly divergent novel proteins. To circumvent the reliance on strict sequence homology, early machine learning methods modeled functional mappings using algorithms such as Support Vector Machines (Cai et al., 2003) and Random Forests (Cai et al., 2003). These approaches integrated heterogeneous hand-crafted descriptors, including physicochemical properties, evolutionary profiles, and protein-protein interaction networks. While establishing valuable baselines, their representational capacity was fundamentally bottlenecked by the manual feature engineering process and a limited ability to resolve highly nonlinear, high-dimensional label dependencies. More recently, deep learning-based methods have substantially improved AFP by learning protein representations in an end-to-end manner. For example, DeepGO (Kulmanov et al., 2018) incorporates neural networks to model GO-related functional dependencies; DeepFRI (Gligorijević et al., 2021) combines sequence and structural information to improve prediction; DeepGOZero (Kulmanov and Hoehndorf, 2022) exploits ontology axioms and embedding-space constraints for better generalization; and TALE (Cao and Shen, 2021) applies Transformer-based modeling to jointly represent protein sequences and hierarchical labels. Building upon these foundations, recent specialized frameworks have emerged to push the performance boundaries by directly tackling label-space modeling and class imbalance. For instance, GDTGO (Zhang et al., 2025a) represents the contemporary state-of-the-art by employing a Graph Convolutional Network (GCN) explicitly on the GO Directed Acyclic Graph (DAG) to capture label topology, coupled with a cross-attention decoder. The technical rationale for selecting GDTGO as our primary comparative baseline is to rigorously test whether a purely sequence-based architecture augmented with textual semantic priors can match or exceed this explicit GCN-based label modeling. Concurrently, other recent works like GOBoost (Zhang et al., 2025b) emphasize the critical necessity of addressing the extreme long-tailed distribution of GO annotations. This background highlights the persistent challenge of rare-label interference during attention-based training and motivates our gradient-decoupling strategy.

Despite these advances, existing methods still leave several key challenges insufficiently addressed. First, a subset of high-performing methods benefits from 3D structural information, contact maps, or graph-based structural representations. However, experimentally resolved structures remain unavailable for many proteins, and predicted structures may introduce additional uncertainty and computational overhead (Lin et al., 2023; Ruff and Pappu, 2021). Therefore, developing models that can effectively capture both local functional motifs and long-range contextual dependencies without relying on explicit structural inputs remains an important challenge. Second, the GO label space exhibits a pronounced long-tailed distribution (Zhang et al., 2025b). In conventional end-to-end multi-label learning frameworks, a shared sequence encoder is required to support prediction across both frequent and rare GO terms. Because rare labels provide limited supervision, their label-specific learning signals may interfere with shared representation learning and weaken generalization, particularly under highly imbalanced multi-label settings. Thus, reducing feature interference between shared sequence representation learning and label-specific prediction remains a critical issue for robust protein function prediction. Third, existing methods often overlook the rich biological semantics embedded within the GO terminology. When constructing label representations, they typically rely on random initialization or static node embeddings, which fail to bridge the semantic gap between textual functional descriptions and protein sequences. This deficiency severely limits the model’s discriminative power for rare functional terms. Therefore, effectively injecting transferable semantic priors to augment label representations is highly desirable.

Motivated by these unresolved limitations, we propose S2DA-GO, a sequence-based model using pre-trained protein language model embeddings and GO textual semantic priors. Importantly, to clarify the scope of our methodology, S2DA-GO is a strictly structure-free framework; it directly models protein functions without requiring explicit 3D structures. The framework comprises a global contextual stream for robust sequence-level representation and a local target-aware stream that captures fine-grained, function-specific residue patterns. To mitigate optimization interference under the long-tailed multi-label distribution, we introduce a Gradient-Decoupled Cross-Attention Module. This module allows label query vectors to retrieve function-relevant local sequence regions while strictly limiting the influence of label-specific local gradients on the shared sequence encoder, thereby improving overall representation robustness. Furthermore, to bridge the semantic gap for sparsely annotated terms, we design a Semantic Prior-Driven Label Query Module (SPLQM). By integrating BioBERT-encoded (Lee et al., 2020) GO textual definitions with learnable residual terms, SPLQM adaptively refines label representations while preserving intrinsic biological semantics. Through this synergistic design, S2DA-GO provides a highly effective and robust solution for the large-scale functional annotation of uncharacterized proteins.

## 2 Materials and methods

### 2.1 Dataset

We evaluated S2DA-GO on the curated benchmark dataset originally introduced by DeepFRI (Gligorijević et al., 2021) and subsequently used in PFresGO (Pan et al., 2023), which comprises 36,641 proteins encompassing functional annotations across all three domains of the GO. To ensure data quality and mitigate homologous bias, we subjected the raw sequences to redundancy reduction utilizing the CD-HIT program (Fu et al., 2012). Considering the extreme sparsity of samples associated with long-tailed labels, we applied a CD-HIT sequence identity threshold of 95%. Following this de-redundancy procedure, the curated dataset was partitioned into training, validation, and test sets at an approximate ratio of 8:1:1. To guarantee the rigorousness and reliability of the performance assessment, the test set was strictly restricted to sequences possessing at least one experimentally validated GO annotation. To rigorously prevent homologous leakage and ensure the absolute credibility of our evaluations in low-homology scenarios, we strictly followed the testing protocol established by the benchmark dataset. Specifically, the test dataset is dynamically partitioned into subsets based on maximum sequence identity thresholds (e.g., 30%, 40%, 50%, 70%) calculated relative to the training set. This stringent isolation mechanism guarantees that when evaluating the model on the ≤30% low-homology subset, there are zero test proteins that share more than 30% sequence identity with any sample in the training corpus. Thus, the performance benchmarks reported in our low-homology evaluations are completely insulated from high-homology bias.

### 2.2 Input features

#### 2.2.1 Protein embedding features

The primary structure of each protein is formulated as a variable-length string comprising the 20 standard amino acids. Given that the vast majority of naturally occurring proteins span fewer than 1,000 residues, we imposed a fixed-length truncation and padding protocol across all input sequences in our experiments. Specifically, proteins exceeding this threshold were truncated to their first 1,000 residues, whereas shorter sequences were extended via zero-padding. To comprehensively capture both local contextual motifs and long-range dependencies within the protein sequences, we leveraged the pre-trained protein language model ProtT5 (Elnaggar et al., 2022) to extract residue-level feature representations. During the feature extraction phase, the parameters of ProtT5 were frozen and explicitly excluded from downstream task training. For an input sequence of length L , ProtT5 encodes it into a residue-level feature matrix $E\in R^{L\times d}$ , where d = 1,024 denotes the feature dimensionality of the final hidden state of ProtT5. The derived representations subsequently serve as the foundational input for the protein sequence branch, facilitating the subsequent local-global joint modeling.

#### 2.2.2 GO term embeddings

To inject label-side semantic priors into the model, we further constructed textual representations for the GO terms. Specifically, for each target GO label, we extracted its standard name and textual definition from the official GO database, concatenating them to formulate a comprehensive term description. Subsequently, this composite text was fed into the pre-trained biomedical language model, BioBERT v1.1 (Lee et al., 2020), from which we extracted the corresponding token representation to serve as the initial semantic embedding for the respective GO term with an initial embedding dimensionality of 768. Because BioBERT is pre-trained on a massive corpus of biomedical literature, the generated term representations effectively encapsulate the intrinsic biological semantics of the GO labels, rendering them highly suitable as static semantic priors on the label side. To enable these priors to further adapt to the downstream protein function prediction task, we applied a linear projection to them within the network and jointly optimized them alongside learnable residual terms. This architectural design bolsters the model’s adaptability to task-specific distributions while preserving the generalized semantic knowledge.

### 2.3 Deep model

#### 2.3.1 Overview of S2DA-GO

**FIGURE 1:**
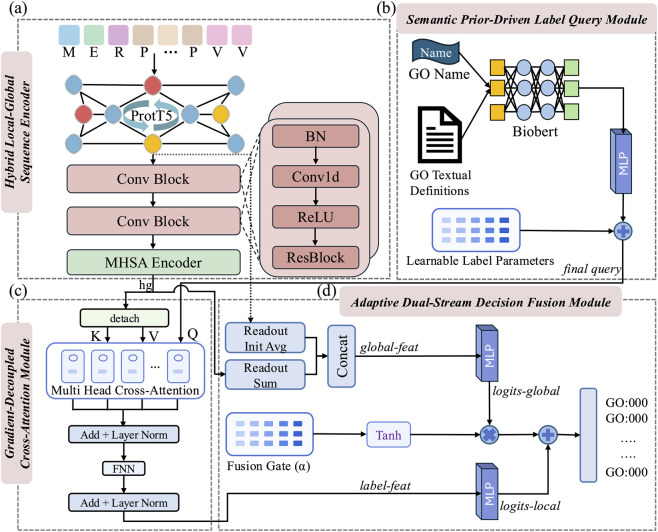
The architecture of S2DA-GO. **(a)** The Hybrid Local-Global Sequence Encoder (HLGSE) ingests 1D amino acid sequences and extracts multi-scale representations by integrating ProtT5 embeddings with convolutional blocks and global self-attention. **(b)** The Semantic Prior-Driven Label Query Module (SPLQM) imports GO textual definitions via BioBERT and transforms them alongside learnable parameters into task-specific semantic queries. **(c)** The Gradient-Decoupled Cross-Attention Module (GDCAM) aligns the semantic queries with the sequence features to isolate function-specific local residue patterns, utilizing a stop-gradient (detach) operation to insulate the shared sequence encoder from label-specific noisy gradients. **(d)** The Adaptive Dual-Stream Decision Fusion Module (ADFM) dynamically integrates the macroscopic global sequence context with the fine-grained local evidence via a learnable fusion gate to generate the final multi-label predictive probabilities.

As shown in Figure 1, S2DA-GO consists of four main modules: the Hybrid Local-Global Sequence Encoder (HLGSE), the SPLQM, the GDCAM, and the Adaptive Dual-Stream Decision Fusion Module (ADFM). To establish a robust feature foundation, the HLGSE first encodes multi-scale patterns by integrating convolutional receptive fields with global self-attention. Recognizing the semantic gap between these sequence features and abstract functional ontologies, the SPLQM injects BioBERT-derived priors to construct informative label-side queries. Building upon these two modalities, the GDCAM facilitates label-aware local interactions through cross-attention; crucially, it incorporates a gradient-decoupling strategy to insulate the shared encoder from the noisy gradients generated by sparse, rare labels. Finally, the ADFM dynamically adjudicates the contributions of global contextual information and local discriminative cues via a learnable gating mechanism, yielding the final predictive logits for protein function annotation.

#### 2.3.2 Hybrid Local-Global Sequence Encoder

Protein functions inherently rely on both locally conserved motifs and long-range inter-residue dependencies (Rives et al., 2021). To comprehensively model these dual-scale informative cues, we designed the HLGSE. By synergistically integrating Convolutional Neural Networks (CNNs) with Transformer encoder layers, this module constructed a hierarchical sequence representation space spanning from local to global receptive fields.

Given an input sequence embedding $X\in R^{L\times d}$ , where L denotes the sequence length and d signifies the feature dimensionality. To provision a stable global baseline representation for the subsequent dual-stream architecture, the HLGSE initially applied Global Average Pooling (GAP) to the raw sequence features, extracting an initial sequence vector $h_{init}\in R^{d}$ , as shown in Equation 1:

$$h_{init}=\frac{1}{L}\sum_{i=1}^{L}X_{i}$$ (1)

Subsequently, the module advanced to the local feature aggregation phase. The network comprised two structurally identical residual convolutional blocks. For the l -th block ( $l\in \left\{1,2\right\}$ ), the forward propagation was formulated as shown in Equations 2, 3, explicitly maintaining consistent channel dimensions and sequence lengths:

$$H_{BN}^{\left(l\right)}=B\text{atch}N\text{orm}\left(H^{\left(l-1\right)}\right)$$ (2)

$$H^{\left(l\right)}=H^{\left(l-1\right)}+\text{Dropout}\left(\text{ReLU}\left(\text{Conv}1D\left(H_{\text{BN}}^{\left(l\right)}\right)\right)\right)$$ (3)

where the convolutional kernel size was set to 3 with a padding of 1, effectively aggregating local contiguous residue information while preserving the temporal dimension. The dropout probability was configured at 0.3 to mitigate the risk of overfitting. Following the two residual convolutional blocks, the locally enhanced sequence representation, denoted as $H_{seq}$ , was fed into a Multi-Head Self-Attention (MHSA) (Vaswani et al., 2017) layer to establish global contextual dependencies. Under a standard Post-Norm configuration, the updating procedure of the Transformer encoder layer is mathematically expressed in Equations 4, 5:

$${\tilde{H}}_{seq}=\text{LayerNorm}\left(H_{seq}+\text{Dropout}\;\left(\text{MHSA}\left(H_{seq},H_{seq},H_{seq}\right)\right)\right)$$ (4)

$$H_{g}=\text{LayerNorm}\left({\tilde{H}}_{seq}+\text{Dropout}\;\left(\text{FFN}\left({\tilde{H}}_{seq}\right)\right)\right)$$ (5)

The number of attention heads in the MHSA was set to 4. Ultimately, the module was output the high-level global representation ${\;H}_{g}$ and the initial baseline feature $h_{\mathrm{init}}$ , which were subsequently utilized for global semantic modeling and dual-stream decision fusion, respectively.

#### 2.3.3 Semantic Prior-Driven Label Query Module

Building upon the static semantic priors $E_{prior}$ derived from BioBERT, this module aimed to transform these raw textual embeddings into task-specific label queries. To bridge the distributional discrepancy between the biomedical text space and the protein sequence space, we introduced a learnable residual mechanism to formulate the final label query matrix $Q_{label}$ , as defined in Equation 6:

$$Q_{label}=Q_{learnable}+{\text{Linear}}_{proj}\left(E_{prior}\right)$$ (6)

where ${\text{Linear}}_{\text{proj}}\left(\cdot \right)$ is a linear projection layer that maps the prior semantic representations into the model’s latent space. $Q_{learnable}\in R^{N\times d}$ denotes the task-related learnable residual parameter matrix, which was initialized to zero.

This semantic prior and learnable residual architecture ensured that the model relied predominantly on stable semantic priors during the nascent stages of training. As optimization proceeded, the learnable residual component gradually assimilated task-specific information, facilitating adaptive refinement of the label query representations. The resulting semantic query matrix $Q_{label}$ was then utilized as the query input for the label-aware cross-attention mechanism in the downstream local branch.

#### 2.3.4 Gradient-decoupled cross-attention module

In the context of large-scale, multi-label protein function annotation, the GO label space inherently exhibits a pronounced long-tailed distribution. If the label-specific loss derived from the local branch were allowed to backpropagate directly through the shared sequence encoder, the unstable gradients engendered by low-frequency and noisy labels could severely disrupt foundational representation learning. To circumvent this detriment, we formulated the GDCAM. This module was meticulously designed to insulate the shared encoder’s optimization trajectory from the local branch’s influence while steadfastly preserving the model’s capacity for local label-aware perception.

Specifically, prior to initiating label-specific interactions within the local branch, we applied a gradient-stopping operation to the high-level sequence representation $H_{g}\in R^{L\times d}$ generated by the HLGSE, yielding the decoupled sequence features, as defined in Equation 7:

$${\tilde{H}}_{g}=\text{StopGradient}\left(H_{g}\right)$$ (7)

This operation ensured that ${\tilde{H}}_{g}$ remains numerically identical to $H_{g}$ during the forward propagation phase, yet it explicitly severed the backward gradient flow from the local branch loss to the underlying sequence encoder. Consequently, the local branch was empowered to learn label-relevant local patterns conditioned on stable sequence representations, whereas the shared backbone encoder remained predominantly optimized via the global branch.

Building upon this decoupled foundation, we assigned the label query matrix $Q_{label}\in R^{N\times d}$ previously derived from the SPLQM, as the Query, while assigning the decoupled sequence features ${\tilde{H}}_{g}\in R^{L\times d}$ as both the Key and Value. These matrices were fed into a Multi-Head Cross-Attention (MHCA) module to explicitly model the spatial correspondence between label semantics and local sequence regions. The computational procedure was formulated as shown in Equations 8, 9:

$$H_{cross}=\text{LayerNorm}\left(Q_{label}+\text{MHCA}\;\left(Q_{label},{\tilde{H}}_{g},{\tilde{H}}_{g}\right)\right)$$ (8)

$$H_{label}=\text{LayerNorm}\left(H_{cross}+\text{FFN}\;\left(H_{cross}\right)\right)$$ (9)

where the number of attention heads in the MHCA was set to be congruent with the multi-head self-attention in the HLGSE, and denoted a Feed-Forward Network (FFN) composed of two linear transformations with a ReLU activation in between, expanding the inner hidden dimension to 2d . Through this sophisticated cross-attention mechanism, each label query vector was guided to adaptively aggregate function-specific local evidence from the sequence feature space via spatial attention weight assignment. This yields the label-aware local representation $H_{label}\in R^{N\times d}$ . Ultimately, $H_{label}$ was routed to the subsequent local branch prediction head to produce label-specific local predictions. By seamlessly integrating gradient decoupling with cross-attention, the GDCAM concurrently amplified the model’s discriminative power for local functional regions and fortified the stability of shared sequence representation learning.

#### 2.3.5 Adaptive Dual-Stream Decision Fusion Module

Following the extraction of global sequence representations and label-aware local representations, we proposed the ADFM to seamlessly integrate macroscopic global context with fine-grained local evidence. In contrast to conventional fusion paradigms such as naive concatenation or fixed-weight summation, the ADFM dynamically orchestrated the two decision streams via a learnable gating parameter, thereby substantially elevating training stability and terminal predictive performance.

Initially, within the global branch, the high-level sequence representation $H_{g}\in R^{L\times d}$ derived from the HLGSE undergoes sum pooling along the sequence dimension. This pooled representation was subsequently concatenated with the initial baseline vector $h_{init}$ along the feature dimension to formulate the comprehensive input for the global branch. The concatenated joint feature was then fed into a Multi-Layer Perceptron (MLP) to yield the global predictive logits, as shown in Equations 10–12:

$$h_{pool}=\sum_{i=1}^{L}H_{g,i}$$ (10)

$$h_{global}=\left[h_{pool}∥h_{init}\right]$$ (11)

$$Y_{global}={\text{MLP}}_{global}\left(h_{global}\right)\in R^{N}$$ (12)

where ∥ denotes the feature concatenation operation, ${\text{MLP}}_{global}$ comprises a stack of fully connected layers equipped with batch normalization, non-linear activations, and dropout regularization. This branch was intrinsically tailored to learn generalizable decision boundaries within the protein functional space.

Concurrently, within the local branch, the label-aware feature matrix $H_{label}\in R^{N\times d}$ generated by the GDCAM was projected into label-level local predictive logits, as defined in Equation 13:

$$Y_{local}={\text{MLP}}_{local}\left(H_{label}\right)\in R^{N}$$ (13)

To elegantly amalgamate these two decision streams, which inherently possess disparate representational granularities, we introduced a learnable scalar gating parameter λ∈R ,The final multi-label predictive distribution $Y_{\mathrm{final}}\in R^{N}$ was thus formulated as shown in Equation 14:

$$Y_{final}=Y_{global}+\tan h\left(\lambda \right)\cdot Y_{local}$$ (14)

where λ was initialized to zero. This strategic initialization dictated that the model predominantly relies on the global branch for predictions during the nascent phases of training. As optimization progressed, the label-specific insights gleaned from the local branch were gradually and adaptively incorporated into the final decision-making process. Through this paradigm, the ADFM preserved the robustness of global predictions while seamlessly infusing complementary discriminative cues derived from local functional regions. Ultimately, $Y_{final}$ was routed to the subsequent activation and loss computation modules to generate the definitive multi-label predictions.

### 2.4 Loss function

Protein function prediction was formulated as an imbalanced multi-label classification task. Since GO annotations exhibit a long-tailed distribution, standard binary cross-entropy may be dominated by abundant easy negative labels. Therefore, we adopted focal loss (Lin et al., 2017) to reduce the contribution of easy labels and emphasize relatively hard or sparsely supervised labels during training. For the i -th GO term, with ground-truth label $y_{i\;}$ and predicted probability ${\tilde{y}}_{i\;}$ , the focal loss was defined as shown in Equation 15:

$$L_{focal}=-\frac{1}{N}\sum_{i=1}^{N}\left[{\left(1-{\tilde{y}}_{i\;}\right)}^{\gamma }y_{i}\log\left({\tilde{y}}_{i\;}\right)+{\tilde{y}}_{i}^{\gamma }\left(1-y_{i}\right)\log\left(1-{\tilde{y}}_{i\;}\right)\right]$$ (15)

where N denotes the total number of GO terms, γ is the focusing parameter that controls the down-weighting of easy examples. When γ=0 , focal loss is equivalent to binary cross-entropy (BCE) loss. Following previous protein function prediction practice, γ was set to two in all experiments.

While S2DA-GO generates raw predictive probabilities for individual GO terms, these initial predictions do not intrinsically guarantee compliance with the topological structure of the Gene Ontology. The GO is structured as a DAG (Valentini, 2011), which is governed by the True Path Rule (TPR): if a protein is annotated with a specific term, it must logically be annotated with all of its ancestral terms in the DAG.

To explicitly enforce this hierarchical dependency relationship and ensure the biological validity of our outputs, we apply a deterministic prediction propagation algorithm as a post-processing step during the inference and evaluation phases. Let ${\tilde{y}}_{i\;}$ denote the raw probability score predicted by the S2DA-GO neural network for the i -th GO term. To satisfy the TPR constraint, the finalized probability score ${\tilde{y}}_{i\;}$ is recalibrated using a bottom-up propagation strategy along the GO DAG. Specifically, the updated score for any parent term is guaranteed to be greater than or equal to the scores of all its descendant terms. This topological constraint is mathematically formulated as shown in Equation 16:

$${\tilde{y}}_{i\;}=\max\left({\tilde{y}}_{i\;},\max_{c\in children\left(i\right)}{\tilde{y}}_{c\;}\right)$$ (16)

where $children\left(i\right)$ represents the set of immediate child nodes of term i in the ontological graph. By recursively applying this operation from the leaf nodes up to the root nodes of the MF, BP, and CC branches, the final predictive distributions strictly adhere to the hierarchical axioms of the GO. All performance metrics reported in our evaluation are calculated based on these DAG-calibrated probabilities.

### 2.5 Implementation and reproducibility

The S2DA-GO framework was implemented in PyTorch and optimized using the AdamW algorithm. To ensure reproducibility while adhering to space constraints, we provide the exhaustive configuration of hyperparameters including the learning rate schedule, focal loss parameters, and the validation-based ensemble strategy along with the detailed mathematical formulations of the evaluation metrics in Section 2 of the Supplementary Material.

## 3 Results

### 3.1 Performance comparison on the benchmark datasets

To evaluate the performance of S2DA-GO, we conducted a comparative analysis against representative baseline and state-of-art methods using the benchmark test set. As summarized in Table 1, S2DA-GO achieved the highest performance across all evaluation metrics and Gene Ontology domains.

**TABLE 1:** Performance comparison between S2DA-GO and state-of-the-art methods on the test set across all three GO domains. Bold numbers denote the best results.

Method	AUPR			Fmax		
	MF	BP	CC	MF	BP	CC
BLAST	13.6%	6.7%	9.7%	32.8%	33.6%	44.8%
DeepGO	39.1%	18.2%	26.3%	57.7%	49.3%	59.4%
FunFams	36.7%	26.0%	28.8%	57.2%	50.0%	62.7%
DeepFRI	49.5%	26.1%	27.4%	62.5%	54.0%	61.3%
TALE+	56.4%	30.2%	32.5%	66.2%	55.4%	61.0%
DeepGOZero	61.4%	29.4%	31.4%	71.9%	56.4%	53.4%
HEAL-PDB	57.1%	25.9%	34.2%	69.1%	56.5%	65.5%
PFresGO	60.2%	29.3%	36.1%	69.2%	56.8%	67.4%
GDTGO	62.6%	31.5%	38.4%	71.0%	57.2%	67.8%
S2DA-GO	**65.1%**	**33.4%**	**41.7%**	**73.1%**	**59.5%**	**68.6%**

Specifically, regarding the AUPR metric, S2DA-GO ranked first among the compared methods in terms of AUPR by consistently outperforming a wide array of baseline categories. The improvement was most evident in the BP branch when compared with earlier methods. Notably, even when evaluated against the latest highly competitive deep learning frameworks such as PFresGO and GDTGO (Zhang et al., 2025a). S2DA-GO still secured substantial relative improvements of 4.0%, 6.0%, and 8.6% in the MF, BP, and CC domains, respectively.

A similar trend was observed for the $F_{\max}$ metric. While earlier models (e.g., DeepFRI, DeepGO) exhibited highly variable performance across different ontological branches, S2DA-GO obtained the highest $F_{\max}$ values in MF, BP, and CC. By achieving $F_{\max}$ scores of 73.1%, 59.5%, and 68.6%, the proposed framework comprehensively surpasses the compared sequence-based and structure-informed methods. Overall, S2DA-GO ranked first among the compared methods for both AUPR and $F_{\max}$ across all three GO domains.

### 3.2 Performance evaluation via Precision-Recall curves

**FIGURE 2:**
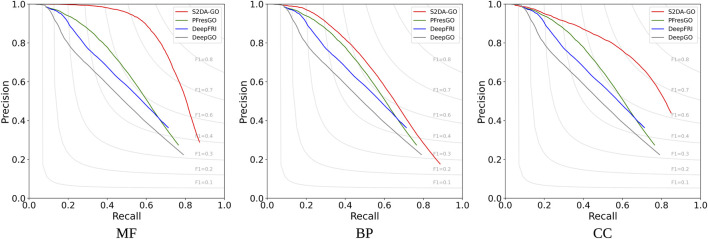
Precision-recall curves of S2DA-GO and other methods on MF, BP and CC terms.

To comprehensively assess the predictive fidelity of the proposed model, we plotted the protein-centric Precision-Recall (PR) curves for S2DA-GO alongside three state-of-the-art baselines (PFresGO, DeepFRI, and DeepGO) across the MF, BP, and CC sub-ontologies (Figure 2). Overall, the PR curves generated by S2DA-GO consistently envelop those of the comparative baselines across all three functional domains.

Specifically, within the MF and CC domains, the PR trajectories of S2DA-GO distinctly gravitate towards the higher $F_{1}$ -score regions, sustaining elevated precision across a broader spectrum of recall rates. By contrast, the precision of PFresGO, DeepFRI, and DeepGO exhibits a more precipitous decline as recall increases. Regarding the challenging BP domain, although the performance margins among all evaluated methods are relatively narrow, the PR curve of S2DA-GO remained above those of the compared methods for most recall values.

### 3.3 Robustness evaluation across varying sequence identities

To evaluate the model’s generalizability concerning novel proteins with low homology relative to the training corpus, we assessed S2DA-GO across test subsets partitioned by varying sequence similarities. The test set was stratified into five distinct subsets based on maximum sequence identity thresholds relative to the training data: 30%, 40%, 50%, 70%, and 95%. Within these subsets, we benchmarked S2DA-GO against DeepGO, DeepFRI, TALE+, HEAL-PDB (Gu et al., 2023), and PFresGO using the AUPR and $F_{\max}$ metrics.

**FIGURE 3:**
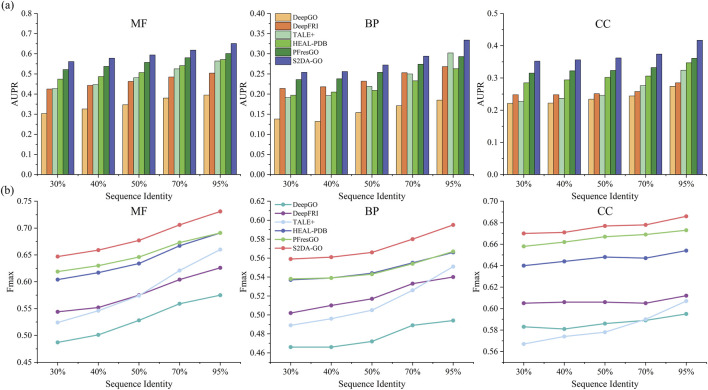
**(a)** AUPR and **(b)** $F_{\max}$ for different methods with varying sequence identities on the test set.

As depicted in Figure 3, the predictive performance of all evaluated models exhibits an upward trajectory concomitant with increasing sequence identity. Nevertheless, S2DA-GO consistently outperforms all baseline methodologies across the three GO domains at every discrete identity threshold. Notably, the performance gap is most pronounced within the subsets characterized by extremely low sequence identity. For instance, in the most stringent less 30% identity subset for the MF domain, S2DA-GO maintained an AUPR of 56.1%, exceeding the second-best method, PFresGO, by 4.0 percentage points. Similar ranking patterns were observed in the other low-identity subsets.

### 3.4 Ablation study

To comprehensively evaluate the specific contributions of individual architectural components within the S2DA-GO framework, we conducted a systematic ablation study. We constructed four ablated variants by disabling specific mechanisms corresponding to our core sub-modules: 1). S2DA-GO W/O Detach: Removed the gradient-stopping operation from the GDCAM. 2). S2DA-GO W/O Gate: Discarded the learnable gating parameter within the ADFM, replacing the adaptive fusion with an unweighted direct summation of the global and local streams. 3). S2DA-GO W/O Biobert: Replaced the BioBERT-encoded GO label semantic priors with randomly initialized vectors. 4). S2DA-GO W/O CrossAttn: Excised the cross-attention layer from the GDCAM. These variants were benchmarked against the intact S2DA-GO model across the MF, BP, and CC sub-ontologies utilizing the AUPR and $F_{\max}$ metrics.

As delineated in Table 2, the complete S2DA-GO model achieved the optimal overall performance, securing the highest scores across all metrics and functional domains. The removal of any single module resulted in a conspicuous degradation in predictive capacity. Analyzing the performance drops reveals specific sensitivities across the GO domains. Notably, excising the cross-attention module (S2DA-GO W/O CrossAttn) precipitated the most significant performance decline in the MF category, reducing the AUPR by nearly 1.0 percentage point (from 65.1% to 64.2%). Conversely, the exclusion of BioBERT semantic priors (S2DA-GO W/O Biobert) severely compromised predictions in the CC category, where the AUPR experienced the steepest drop among all variants (from 41.7% to 39.9%). Furthermore, discarding the gating mechanism (S2DA-GO W/O Gate) disproportionately affected the BP category, resulting in maximum reductions in both AUPR (down 1.2 percentage points) and $F_{\max}$ (down 0.9 percentage points). Finally, the S2DA-GO W/O Detach variant exhibited a pervasive, sub-optimal performance across all three domains compared to the full model. Overall, removing individual components led to domain-specific changes in AUPR and $F_{\max}$ .

**TABLE 2:** Ablation experiment results of S2DA-GO on the test set. Bold numbers denote the best results.

Method	AUPR			Fmax		
	MF	BP	CC	MF	BP	CC
S2DA-GO W/O Detach	64.9%	33.1%	40.5%	73.0%	59.2%	67.4%
S2DA-GO W/O Gate	64.6%	32.2%	41.2%	72.1%	58.6%	68.2%
S2DA-GO W/O BioBERT	64.9%	33.0%	39.9%	72.9%	59.2%	67.2%
S2DA-GO W/O CrossAttn	64.2%	32.7%	40.6%	73.1%	58.9%	68.1%
S2DA-GO	**65.1%**	**33.4%**	**41.7%**	**73.1%**	**59.5%**	**68.6%**

### 3.5 S2DA-GO localizes function-associated residues

Given that spatially clustered functional residues are pivotal for protein function, we mapped the learned attention weights onto raw amino acid sequences and their corresponding 3D structures. These highly weighted regions were then benchmarked against experimentally validated ligand-binding sites curated in the BioLiP database (Yang et al., 2012). Here, we detail two representative case studies: PDB:3D84, chain X (Cody et al., 2008) and PDB:1KV5, chain A (Kursula et al., 2002).

**FIGURE 4:**
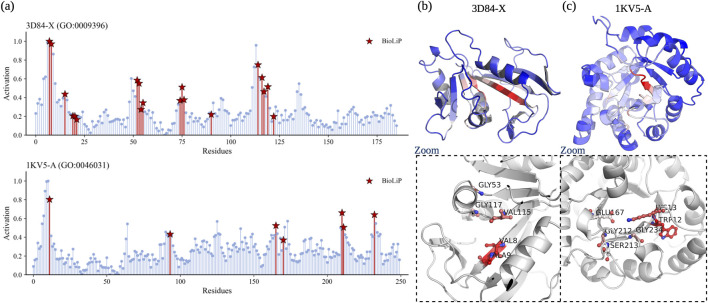
S2DA-GO locates functional residues based on residue-level attention weights. **(a)** The functional site prediction results for two representative proteins (PDB: 3D84-X, Chain X; PDB:1KV5, Chain A) each associated with distinct functional annotations (GO:0009396; GO:0046031), the blue lollipop plot represents the attention weights assigned by the model, while the red stars indicate experimentally annotated functional residues obtained from the BioLiP database. **(b,c)** Tertiary structures for two representative proteins, with structural renderings highlighting residue-level attention weights.

For the protein 3D84-X, the 1D attention weight distribution exhibits distinct activation peaks across the sequence (Figure 4a). The residues corresponding to these maximum weights align precisely with the ground-truth functional binding sites documented in BioLiP. Upon projecting these 1D attention weights onto the 3D crystal structure (Figure 4b), it is observed that these highly activated residues (highlighted in red) cluster in three-dimensional space to form a well-defined binding pocket.

We further analyzed the protein 1KV5-A, which achieved an AUROC score of 0.9489 for the prediction of the GO term GO:0046031 (see Supplementary Figure S1 for detailed ROC curves). This target contains exactly seven active binding sites distributed across a 250-residue sequence. As depicted in the refined 3D rendering (Figure 4c), the model assigned dominant attention weights to the genuine binding residues. Notably, although these identified residues are separated by vast distances along the 1D primary sequence, they converge spatially in the folded protein. The spatial mapping further showed that several high-attention residues were close in the folded structures. Overall, the qualitative structural localizations observe that S2DA-GO is capable of recovering spatially contiguous functional residues strictly from one-dimensional sequence inputs and semantic priors. Further methodological interpretations and limitations of these residue-level attention patterns are detailed in the Discussion section.

## 4 Discussion

The present study addressed two major challenges in protein function prediction: modeling both local function-related sequence patterns and global contextual dependencies without relying on explicit structural inputs and improving representation learning under the long-tailed GO label distribution. To tackle these limitations, we developed S2DA-GO, a structure-free framework operating solely on sequence representations and GO semantic priors. Empirical evaluations confirm that coupling local-global sequence extraction with gradient-decoupled cross-attention and adaptive decision fusion enables the model to achieve consistent performance gains across the MF, BP, and CC domains.

The performance gains of S2DA-GO may be related to several architectural designs. Of primary importance, the HLGSE combines 1D convolutional layers with multi-head self-attention, allowing the model to capture both short-range residue patterns and long-range sequence dependencies. Of secondary importance, the SPLQM incorporates BioBERT-derived textual representations of GO terms into the label representation space, which may provide a more informative initialization for low-frequency GO terms than purely random label embeddings. Lastly, the GDCAM enables label queries to interact with residue-level sequence representations and retrieve label-specific local evidence. By applying a stop-gradient operation in the local branch, GDCAM limits the back-propagation of local label-specific gradients into the shared sequence encoder, which may reduce potential interference between local label-specific learning and shared sequence representation learning.

**FIGURE 5:**
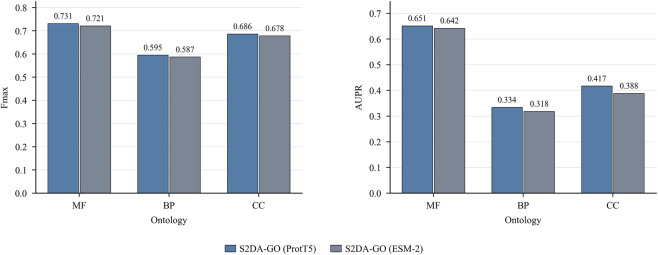
Performance comparison of the S2DA-GO architecture across different Protein Language Models (PLMs). The bar charts illustrate the $F_{\max}$ (left) and AUPR (right) scores of the framework utilizing ProtT5 (S2DA-GO (ProtT5)) versus ESM-2 (S2DA-GO (ESM-2)) as the foundational sequence feature extractor across the MF, BP, and CC ontologies. The consistent performance across both metrics demonstrates that the architectural improvements of S2DA-GO are robust and fundamentally PLM-agnostic.

Beyond the primary evaluations utilizing ProtT5, it is crucial to verify that the performance gains of S2DA-GO are intrinsically derived from its architectural innovations rather than being overly reliant on a specific Protein Language Model (PLM). To demonstrate the generalizability of our framework, we conducted supplementary experiments substituting ProtT5 with ESM-2 (ESM-2–650M) last hidden layer as the foundational feature extractor within the HLGSE module (Figure 5). The empirical results confirm that the S2DA-GO architecture maintains highly stable performance under the ESM-2 feature space. Specifically, S2DA-GO (ESM-2) achieved robust AUPR scores of 64.2%, 31.8%, and 38.8%, alongside $F_{\max}$ scores of 72.1%, 58.7%, and 67.8% across the MF, BP, and CC domains, respectively. While these metrics exhibit only a nominal decrease compared to the ProtT5-based implementation, they continue to consistently outperform the previously compared baseline models. By synergistically integrating feature extraction, sequence-label alignment, and gradient insulation, the framework effectively translates generalized pre-trained embeddings into highly discriminative functional spaces, demonstrating its adaptability for sequence-to-function mapping.

In addition to this architectural adaptability across different PLMs, the results under varying sequence identity thresholds further demonstrate that S2DA-GO does not merely rely on high sequence similarity to the training set. Although the performance of most methods improved as the sequence identity threshold increased, S2DA-GO maintained higher performance than the compared methods across the evaluated thresholds, including low-identity subsets. One possible explanation is that the model benefits from the combination of local sequence motifs, global contextual representations, and label-side semantic priors, rather than relying solely on sequence similarity. This property may be useful for annotating proteins with limited homology to previously characterized proteins.

Compared with existing methods, S2DA-GO has two main methodological distinctions. First, unlike methods that mainly emphasize inter-label relationships or standard sequence-label attention, S2DA-GO introduces gradient decoupling to limit the influence of local label-specific gradients on the shared sequence encoder. This provides an optimization-oriented perspective for long-tailed multi-label protein function prediction. Second, compared with structure-dependent methods such as DeepFRI or HEAL-PDB, S2DA-GO is a structure-free sequence-based framework that does not require explicit 3D structures, contact maps, residue-contact graphs, or protein-protein interaction networks. Therefore, this sequence-based model using pre-trained protein language model embeddings and GO textual semantic priors provides a highly scalable alternative for large-scale protein function annotation, especially when experimentally resolved structures or reliable predicted structures are unavailable.

Beyond predictive performance, the residue-level attention analyses suggest that S2DA-GO can provide interpretable attention patterns in selected proteins. In the case studies, high-attention residues overlapped with BioLiP-annotated ligand-binding sites, and several highlighted residues were spatially close in the corresponding 3D structures. These observations suggest that the label-aware local branch may help generate residue-level hypotheses related to functional regions (Wiegreffe and Pinter, 2019). Looking forward, the S2DA-GO framework opens several promising avenues for future research. While the current architecture demonstrates exceptional efficacy utilizing predominantly 1D sequences and semantic priors, future iterations could naturally integrate evolutionary information, such as multiple sequence alignments (MSAs) or profile-based features (Remmert et al., 2012; Steinegger and Söding, 2017). This extension would further enhance the model’s capacity to delineate subtle functional discrepancies among highly similar paralogous proteins. Additionally, extending this framework to evaluate zero-shot prediction for completely unseen GO terms presents an exciting opportunity to address extreme label sparsity beyond the predefined benchmark vocabularies. Finally, conducting large-scale, systematic quantitative validations of the attention-based residue localization against experimental databases will further solidify the biological interpretability of the learned attention patterns.

In summary, these findings directly address the research questions raised in the introduction. Supported by both quantitative performance benchmarks and qualitative structural localizations, our results indicate that the strategic combination of GO semantic priors with gradient-decoupled label-aware modeling fundamentally improves representation learning and label discrimination in imbalanced multi-label settings. Without relying on explicit 3D structural inputs, the synergistic integration of local-global sequence encoding, gradient-decoupled cross-attention, and adaptive dual-stream fusion substantially enhances predictive performance, remaining robust even within subsets characterized by low sequence identity. Methodologically, S2DA-GO highlights the immense potential of integrating label semantic guidance, local functional region mining, and optimization trajectory control. From a practical perspective, the framework offers a robust, scalable computational approach for the large-scale functional screening of uncharacterized proteins, particularly those sharing limited homology with annotated databases.

## Data Availability

Publicly available datasets were analyzed in this study. This data can be found here: https://github.com/geneticodelab/S2DA-GO.git
