# Supplementary material for: S2DA-GO: enhancing protein function prediction via gradient-decoupled cross-attention and semantic priors
Source: Front Genet. 2026 Jul 9;17:1880976. doi: 10.3389/fgene.2026.1880976 (PMC13391045; doi:10.3389/fgene.2026.1880976)
Supplement: Supplementary file 1 [file DataSheet1.docx]

Supplementary Material

**1. Baseline Model**

**Supplementary Table 1. Summary of the baseline models for protein function annotation.**

| Tool | Year | Feature encoding scheme | Algorithm | Evaluation metrics | Input type |
| --- | --- | --- | --- | --- | --- |
| BLAST | 2006 | Sequence similarity | local alignment heuristic | AUPR, $F_{\max}$ | Sequence-only |
| FunFams | 2015 | HMM | HCA | $F_{\max}$ | Sequence-only |
| DeepGO | 2017 | word embedding | CNN | $F_{\max}$ | Sequence-only |
| DeepFRI | 2021 | Contact Map | GCN,  LSTM | AUPR, $F_{\max}$ | Structure-informed |
| TALE+ | 2021 | Sequence embedding | Transformer | AUPR, $F_{\max}$ | Sequence-only |
| DeepGOZero | 2022 | EL Embeddings | MLP | AUPR, $F_{\max}$, $S_{\min}$, AUC | Sequence-only |
| PFresGO | 2023 | One-hot, ProtT5, Anc2vec | DNN | AUPR, $F_{\max}$, $S_{\min}$, AUC | Sequence-only |
| HEAL | 2024 | One-hot, ESM-1b | GCN, Transformer | AUPR, $F_{\max}$, $S_{\min}$ | Structure-informed |
| GDTGO | 2025 | ESM-2, ProtT5, Anc2vec | GCN, Transformer | AUPR, Fmax | Sequence-only |

Abbreviations and Notes:

1. Feature encoding scheme:

HMM: Hidden Markov Model

EL: Embeddings from Language models (often used in ontology embedding)

ESM-1b / ESM-2: Evolutionary Scale Modeling 1b / 2 (Protein language models)

ProtT5: Protein sequence embedding model based on T5 architecture

Anc2vec: Ontology representation learning method capturing hierarchical ancestor relations

1. Algorithm:

HCA: Hierarchical Clustering Analysis

CNN: Convolutional Neural Network

GCN: Graph Convolutional Network

LSTM: Long Short-Term Memory

MLP: Multi-Layer Perceptron

DNN: Deep Neural Network

1. Evaluation metrics:

AUPR: Area Under the Precision-Recall curve

AUC: Area Under the receiver operating characteristic Curve (AUROC)

$F_{max}$: Maximum protein-centric F-score

$S_{min}$: Minimum semantic distance based on information content

**2. Model Training and Evaluation**

The S2DA-GO framework was implemented utilizing the PyTorch deep learning library. To maintain architectural consistency, the hidden dimensionality of the backbone was uniformly set to 1024, and the number of heads in the multi-head attention mechanisms was fixed at 4. Considering the pronounced long-tailed distribution and the extreme positive-negative sample imbalance inherent to the GO label space, we employed the Focal Loss function during the training phase. The focusing parameter, $\gamma$ was configured to 2.0 to amplify the model's learning capacity for low-frequency functional labels.

For model optimization, we utilized the AdamW optimizer with an initial learning rate of 1×10⁻⁴ and a batch size of 64. The models were trained independently across the three distinct GO domains (MF, BP, and CC), with each training process spanning 12 epochs. To bolster predictive robustness and mitigate the variance associated with any single checkpoint, we adopted a validation-performance-based ensemble strategy during the inference phase. Specifically, the predictions from the top 3 epochs were averaged to formulate the final prediction. All computational experiments were executed on a single NVIDIA RTX 4090 GPU equipped with 24 GB of VRAM.

Model performance was evaluated using $F_{max}$ and macro-averaged Area Under the Precision-Recall Curve (macro-AUPR). $F_{max}$ a standard metric widely used in the Critical Assessment of Function Annotation (CAFA) challenges and quantifies the optimal balance between precision and recall across different decision thresholds. We calculated $F_{max}$ according to the CAFA-style protein-centric definition:

$$F_{max}= \max_{t} \left\{ \frac{2\cdot pr\left( t \right)\cdot rc\left( t \right)}{pr\left( t \right)+rc\left( t \right)} \right\}$$

where $pr\left( t \right)$ and $rc\left( t \right)$denote the average precision and recall at threshold t$\in\left[ 0,1 \right]$ respectively. These are defined as：

$$pr\left( t \right)= \frac{1}{m\left( t \right)}\cdot\sum_{i =1}^{m\left( t \right)} {pr}_{i}\left( t \right)$$

$$rc\left( t \right)= \frac{1}{N}\cdot\sum_{i =1}^{N} {rc}_{i}\left( t \right)$$

where, $m\left( t \right)$represents the number of proteins for which at least one functional term is predicted with a confidence score greater than or equal to $t$, and $N$ is the total number of proteins in the target dataset. The terms $pr_{i}\left( t \right)$ and $rc_{i}\left( t \right)$ correspond to the precision and recall for a specific protein $i$ at threshold $t$, computed as follows：

$${pr}_{i}\left( t \right)=\frac{\sum_{f} I\left( f\in P_{i}\left( t \right)\bigwedge f\in T_{i} \right)}{\sum_{f} I\left( f\in P_{i}\left( t \right) \right)}$$

$${rc}_{i}\left( t \right)=\frac{\sum_{f} I\left( f\in P_{i}\left( t \right)\bigwedge f\in T_{i} \right)}{\sum_{f} I\left( f\in T_{i} \right)}$$

where $P_{i}\left( t \right)$ denotes the set of GO labels predicted for the $i$-th protein at threshold $t$, and $T_{i}$ represents the ground-truth set of true GO labels for that protein. $I\left( \cdot\right)$is the standard indicator function, yielding 1 if the condition is true and 0 otherwise.

In addition to the aforementioned metrics, we evaluate our model using the macro-averaged Area Under the Precision-Recall Curve (macro-AUPR) and the Area Under the Receiver Operating Characteristic Curve (AUROC). Given that protein function prediction is a highly imbalanced multi-label task, macro-AUPR is particularly advantageous because it strictly penalizes false positives and treats all classes equally. Specifically, macro-AUPR is calculated by first computing the individual AUPR for each GO term and then averaging these values across all terms within each respective ontology. Meanwhile, AUROC assesses the model's overall capacity to differentiate between positive and negative instances, such as predicting whether a protein is linked to a given GO term.

**Supplementary Table 2.** Statistical summary of the training and test datasets curated in this study.

| Data | MF | BP | CC |
| --- | --- | --- | --- |
| Train | 29902 | 29902 | 29902 |
| Validation | 3323 | 3323 | 3323 |
| Test | 3416 | 3416 | 3416 |
| GO terms | 489 | 1943 | 320 |


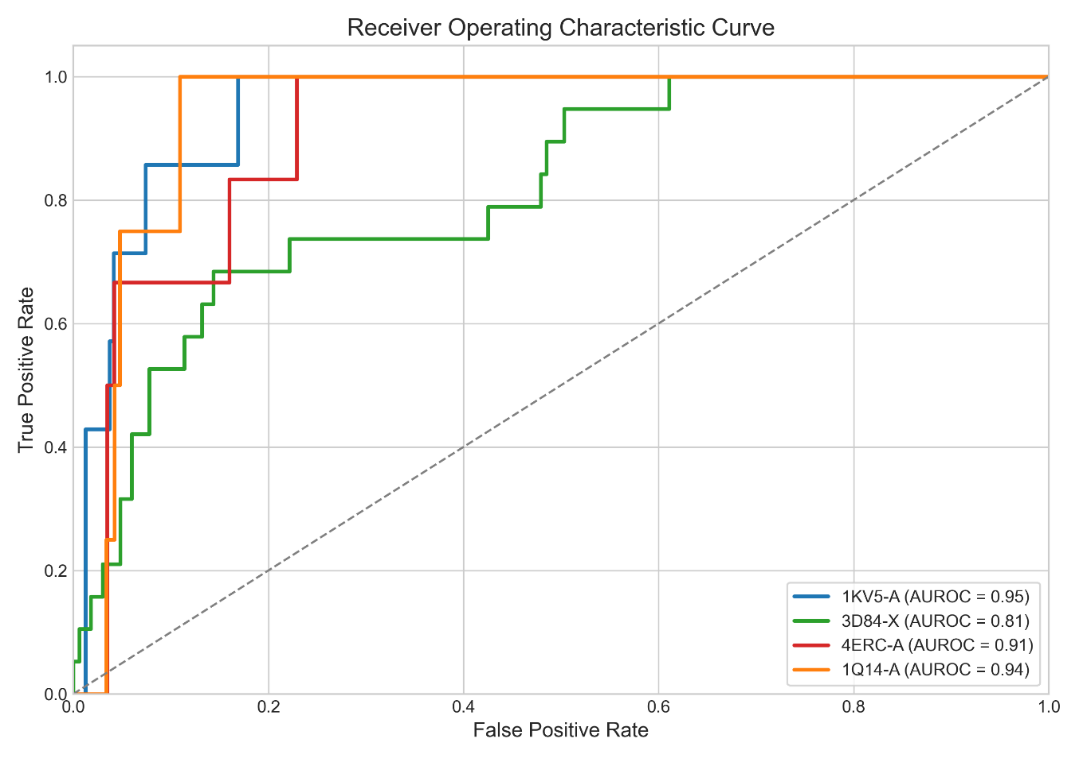


**Supplementary Figure 1.** ROC curves of residues identified by attention weights.

**Supplementary Table 3.** Number of the test set proteins under different sequence identity thresholds to the training set.

| threshold | 30% | 40% | 50% | 70% | 95% |
| --- | --- | --- | --- | --- | --- |
| Proteins | 1716 | 1936 | 2198 | 2732 | 3416 |

**Supplementary Table 4.** AUPR of S2DA-GO and other methods on the test set at the five homology thresholds.

| Model | | | AUPR of MF ontology | | | | | | | | | | | |
| --- | --- | --- | --- | --- | --- | --- | --- | --- | --- | --- | --- | --- | --- | --- |
|  |  |  | <30% | | <40% | | | <50% | | | <70% | | | <95% |
| DeepGO | | | 0.303 | | 0.326 | | | 0.347 | | | 0.38 | | | 0.395 |
| DeepFRI | | | 0.425 | | 0.443 | | | 0.463 | | | 0.485 | | | 0.504 |
| TALE+ | | | 0.427 | | 0.447 | | | 0.481 | | | 0.525 | | | 0.564 |
| HEAL-PDB | | | 0.474 | | 0.487 | | | 0.507 | | | 0.541 | | | 0.571 |
| PFresGO | | | 0.521 | | 0.537 | | | 0.557 | | | 0.580 | | | 0.601 |
| S2DA-GO | | | **0.561** | | **0.578** | | | **0.594** | | | **0.618** | | | **0.651** |
| Model | | | AUPR of BP ontology | | | | | | | | | | | |
|  |  |  | <30% | | <40% | | | <50% | | | <70% | | | <95% |
| DeepGO | | 0.138 | | | 0.132 | | 0.154 | | | 0.171 | | | 0.185 | |
| DeepFRI | | 0.214 | | | 0.218 | | 0.232 | | | 0.253 | | | 0.268 | |
| TALE+ | | 0.191 | | | 0.196 | | 0.219 | | | 0.250 | | | 0.302 | |
| HEAL-PDB | | 0.197 | | | 0.205 | | 0.209 | | | 0.233 | | | 0.263 | |
| PFresGO | | 0.236 | | | 0.238 | | 0.254 | | | 0.274 | | | 0.293 | |
| S2DA-GO | | **0.254** | | | **0.256** | | **0.272** | | | **0.294** | | | **0.334** | |
| Model | | AUPR of CC ontology | | | | | | | | | | | | |
|  |  | <30% | | | <40% | | <50% | | | <70% | | | <95% | |
| DeepGO | 0.221 | | | 0.222 | | 0.234 | | | 0.244 | | | 0.274 | | |
| DeepFRI | 0.248 | | | 0.248 | | 0.251 | | | 0.258 | | | 0.285 | | |
| TALE+ | 0.227 | | | 0.237 | | 0.246 | | | 0.276 | | | 0.324 | | |
| HEAL-PDB | 0.285 | | | 0.294 | | 0.302 | | | 0.306 | | | 0.347 | | |
| PFresGO | 0.315 | | | 0.322 | | 0.323 | | | 0.332 | | | 0.361 | | |
| S2DA -GO | **0.352** | | | **0.356** | | **0.362** | | | **0.374** | | | **0.417** | | |

**Supplementary Table 5.** Fmax of S2DA-GO and other methods on the test set at five homology threholds.

| Model | | $\boldsymbol{F}_{\boldsymbol{max}}$ of MF ontology | | | | | | | | |
| --- | --- | --- | --- | --- | --- | --- | --- | --- | --- | --- |
|  |  | <30% | <40% | | <50% | | <70% | | <95% | |
| DeepGO | | 0.487 | 0.501 | | 0.528 | | 0.559 | | 0.575 | |
| DeepFRI | | 0.544 | 0.552 | | 0.575 | | 0.604 | | 0.626 | |
| TALE+ | | 0.524 | 0.546 | | 0.574 | | 0.621 | | 0.660 | |
| HEAL-PDB | | 0.604 | 0.617 | | 0.634 | | 0.667 | | 0.691 | |
| PFresGO | | 0.619 | 0.630 | | 0.646 | | 0.673 | | 0.691 | |
| S2DA -GO | | **0.647** | **0.659** | | **0.677** | | **0.706** | | **0.731** | |
| Model | | $\boldsymbol{F}_{\boldsymbol{max}}$ of BP ontology | | | | | | | | |
|  |  | <30% | <40% | | <50% | | <70% | | <95% | |
| DeepGO | | 0.466 | 0.466 | | 0.472 | | 0.489 | | 0.494 | |
| DeepFRI | | 0.502 | 0.510 | | 0.517 | | 0.533 | | 0.540 | |
| TALE+ | | 0.489 | 0.496 | | 0.505 | | 0.526 | | 0.551 | |
| HEAL-PDB | | 0.537 | 0.539 | | 0.544 | | 0.555 | | 0.566 | |
| PFresGO | | 0.538 | 0.539 | | 0.543 | | 0.554 | | 0.567 | |
| S2DA-GO | | **0.559** | **0.561** | | **0.566** | | **0.580** | | **0.595** | |
| Model | | $\boldsymbol{F}_{\boldsymbol{max}}$ of CC ontology | | | | | | | | |
|  |  | <30% | <40% | | <50% | | <70% | | <95% | |
| DeepGO | 0.583 | | | 0.581 | | 0.586 | | 0.589 | | 0.595 |
| DeepFRI | 0.605 | | | 0.606 | | 0.606 | | 0.605 | | 0.612 |
| TALE+ | 0.567 | | | 0.574 | | 0.578 | | 0.590 | | 0.607 |
| HEAL-PDB | 0.640 | | | 0.644 | | 0.648 | | 0.647 | | 0.654 |
| PFresGO | 0.658 | | | 0.662 | | 0.667 | | 0.669 | | 0.673 |
| S2DA-GO | **0.670** | | | **0.671** | | **0.677** | | **0.678** | | **0.686** |
